# The impact of redesigning care processes on quality of care: a systematic review

**DOI:** 10.1186/s12913-016-1266-0

**Published:** 2016-01-19

**Authors:** Janneke E. van Leijen-Zeelenberg, Arianne M. J. Elissen, Kerstin Grube, Arno J. A. van Raak, Hubertus J. M. Vrijhoef, Bernd Kremer, Dirk Ruwaard

**Affiliations:** 1 Department of Health Services Research, School for Public Health and Primary Care (CAPHRI), Maastricht University, Maastricht, The Netherlands; 2 Faculty of Health, Medicine and Life Sciences, Maastricht University, Maastricht, The Netherlands; 3 Saw Swee Hock School of Public Health, National University of Singapore, Singapore, Singapore; 4 Scientific Center of Care and Welfare (Tranzo), Tilburg University, Tilburg, The Netherlands; 5 Department of Family Medicine, Free University of Brussels, Brussels, Belgium; 6 Department of Otorhinolaryngology, Head and Neck Surgery, Maastricht University Medical Center, Maastricht, The Netherlands

**Keywords:** Process redesign, Quality of care, Healthcare processes, Systematic review

## Abstract

**Background:**

This literature review evaluates the current state of knowledge about the impact of process redesign on the quality of healthcare.

**Methods:**

Pubmed, CINAHL, Web of Science and Business Premier Source were searched for relevant studies published in the last ten years [2004–2014]. To be included, studies had to be original research, published in English with a before-and-after study design, and be focused on changes in healthcare processes and quality of care. Studies that met the inclusion criteria were independently assessed for excellence in reporting by three reviewers using the SQUIRE checklist. Data was extracted using a framework developed for this review.

**Results:**

Reporting adequacy varied across the studies. Process redesign interventions were diverse, and none of the studies described their effects on all dimensions of quality defined by the Institute of Medicine.

**Conclusions:**

The results of this systematic literature review suggests that process redesign interventions have positive effects on certain aspects of quality. However, the full impact cannot be determined on the basis of the literature. A wide range of outcome measures were used, and research methods were limited. This review demonstrates the need for further investigation of the impact of redesign interventions on the quality of healthcare.

## Background

Growing expenditure on healthcare and ongoing efforts to improve services give impetus to change in processes and systems [1]. As life expectancy increases, so does chronic disease, which is associated with a greater demand for multidisciplinary care [2, 3]. At the same time, public outlay on healthcare has decreased, inducing potential shortages of healthcare providers [3]. Long-term implications for the quality of care are unclear and should be carefully monitored [3]. According to the Institute of Medicine (IoM), patients do not always receive the most suitable care, at the best time or the best place [2]. Its influential report ‘Crossing the Quality Chasm: A New Health System for the 21^st^ Century’ emphasized the need to redesign healthcare processes and systems in response to this quality gap. It called upon providers to ensure more efficient, safe, timely, effective, patient-centered and equitable care [2, 4].

Although some initiatives were undertaken before 2001, the publication of the IoM report served as a catalyst [2, 5]. Numerous interventions – disease management programs for the chronically ill, quality improvement collaboratives, and change programs – are tested and implemented annually on different scales and within different settings [5]. Nonetheless, progress is slow; evaluations of initiatives are inconsistent and available knowledge fragmented [5]. The effects are not homogeneous and the research designs used to measure them are generally weak [4, 6, 7].

This study seeks to establish, through a review of the literature, what is known about the influence of redesigning healthcare processes on the quality of care delivered in the last ten years. Its specific aims are to report (a) the content of the interventions (their objectives and implementation methods); (b) the characteristics of the redesign investigations (study design and setting); and (c) the outcomes on quality of care (patient safety, effectiveness, efficiency, patient-centeredness, timeliness, and equitability). The objective of this literature review is to summarize the current state of knowledge on redesigning healthcare processes and present an overview of improvement efforts in the field.

The review applies several key concepts. The first is ‘process redesign’, defined as any methodology that focuses on creating new processes or changing existing ones in major ways [8]. That definition is deliberately broad so as to cover as many interventions as possible; recourse to dedicated design concepts – such as ‘lean thinking’, ‘business process re-engineering’ or ‘six sigma’ – might exclude relevant studies. The second is ‘quality of care’, connoting healthcare that is safe, effective, patient-centered, timely, efficient and equitable [2]. The third is ‘healthcare processes’, defined as “the activities that constitute healthcare – including diagnosis, treatment, rehabilitation, prevention, and patient education – usually carried out by professional personnel, but also including other contributions to care, particularly by patients and their families”([9], p. 46).

## Methods

### Information sources and search strategy

The search strategy was guided by the PRISMA statement [10]. It was designed to access published work and comprised two stages:

1. An extensive search in Pubmed, CINAHL, Business Source Premier and Web of Science, using predefined search terms and free-text words;
2. A search of the reference lists in the included full-text articles.

From March 2014 through April 2014, the databases PubMed, CINAHL, Web of Science and Business Premier Source (EBSCO-host) were searched by one reviewer (JvL). In PubMed, MeSH terms were used; CINAHL Heading terms were used for CINAHL; and Thesaurus terms were used for Business Premier Source. For Web of Science no predefined keywords were available. Additionally, free-text words were used for all databases. An overview of the search terms is given in Appendix 1.

The database search was limited to articles published in English between January 2004 and April 2014. Articles were included if they presented original research on redesign of healthcare processes, quality of care, and if they assessed the same outcome measures before and after an intervention. (See Table 1 for inclusion and exclusion criteria). Three reviewers (JvL, KG & AE) independently screened titles and abstracts for relevance. The reviewers then held a consensus meeting on the inclusion of articles. When that did not yield agreement, the full text was reviewed and discussed to arrive at a decision. Subsequently, reference lists and bibliographies of all included full-text articles from the first stage were searched for additional studies.

**Table 1:** Inclusion and exclusion criteria

Inclusion criteria	Exclusion criteria
Participants: organizations with a primary focus on healthcare provision	Articles published before 2003
Intervention: either changes in or redesigns of processes in healthcare organizations or healthcare innovations with a clearly described objective to improve quality of care	Articles in which the intervention, data collection methods, data analysis or research context is not described
Outcome measures: quality of care, changeability, process efficiency, patient satisfaction, employee satisfaction, costs of care, facilitators or barriers to implementation, equity, timeliness of care, patient safety, effectiveness.	Articles published in languages other than English.
Outcome measures should be clearly described and be consistent before and after intervention	
Types of studies: RCTs, controlled before-and-after studies, before-and-after studies, interrupted time series, case studies (if using before-and-after measures), mixed methods studies (if using before-and-after measures), observational studies (if using before-and-after measures)	Articles without abstract, articles without before-and-after measurement
	Editorials, viewpoints, non-articles, interviews

### Critical appraisal

Studies meeting the criteria were assessed independently for reporting excellence by three reviewers (JvL, AE & KG), prior to inclusion in light of the Standards for Quality Improvement Reporting Excellence (SQUIRE). That checklist provides guidelines for reporting of studies assessing the effectiveness of interventions to improve quality and safety of care. Its 19 items comprise 38 components [11]. Any disagreements between reviewers were resolved through consensus.

### Data extraction and analysis

After compliance with the reporting guidelines had been assessed, data were extracted independently by three reviewers (JvL, KG & AE) from the results and discussion/conclusion sections. For that purpose, a form was developed. The form contained variables such as publication year, study objectives, characteristics of the redesign and outcome measures. Any disagreements were resolved through consensus. Meta-analysis could not be performed because the studies used different outcome measures and research designs.

## Results

**Fig. 1:**
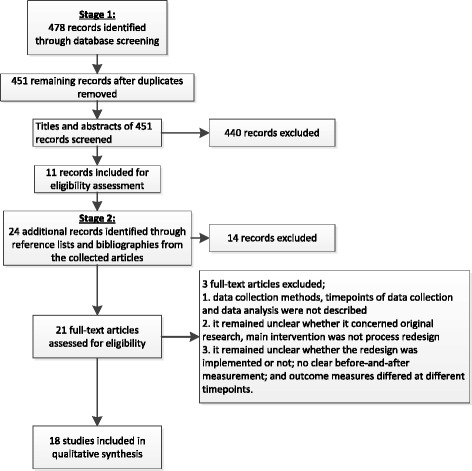
Search strategy

Figure 1 shows the steps leading to inclusion in the review. Initially, after removing duplicates (N = 27), 451 articles were found in the first stage, 11 of which were then included on the basis of their titles and abstracts. Perusal of their reference lists yielded another 24 articles for screening of title and abstract. Based on titles and abstracts, 21 articles were assessed for eligibility. On eight of these, consensus was only reached after reviewing the full text. After assessing the reporting excellence, three articles were excluded. One was removed because it did not describe data collection and timepoints, so it could not be determined whether a before-and-after measurement was performed. Another was removed because it was unclear whether it concerned original research; moreover, the main intervention (presence of a nurse coordinator) did not qualify as process redesign. The third was removed because it was unclear whether the intervention was actually implemented and whether before-and-after measurement was carried out but also because the outcome measures differed at various timepoints. In total, 18 articles were included in the final review.

### Reporting excellence

Table 2 summarizes the findings according to SQUIRE guidelines. The number of components described range from 11 [12] to 27 [13], with most articles reporting on 20 or more [13–22]. Overall, methods of evaluation and analysis are the least well described. The majority described the research setting (N = 16) [12–27], intervention components and parts (N = 16) [13–16, 18–28], main factors in the choice of intervention (N = 15) [11, 13–18, 20, 22–28], and primary and secondary outcomes (N = 15) [12–14, 16–24, 28, 29]. Thirteen articles presented evidence on the strength of the association between the intervention and changes observed (N = 13) [12, 13, 16–22, 24, 25, 27–29]. Half gave details on the qualitative and quantitative methods applied (N = 9) [13, 17–20, 24, 25, 28, 29] or aligned the unit of analysis with the intervention (N = 9) [13–15, 18–21, 24, 28]. Six described internal and external validity [13, 15, 17–20, 28], whereas two dealt with the validity and reliability of instruments [17, 28]. Whereas none of the articles explicitly stated the study questions, all of them specified the aims of the intervention. Most data concerned changes observed in the care delivery process (N = 12) [13–16, 18, 21–24, 26, 28, 29] or differences in patient outcomes (N = 12) [13, 16–24, 28, 29].

**Table 2:** Overview of reporting excellence according to the SQUIRE guidelines

Reference	Introduction	Methods			Results		Conclusion & discussion	Total # SQUIRE components mentioned
		Intervention	Methods of evaluation	Analysis	Setting	Changes in process		
1. Pennell, et al. (2005)	Describes 4/5 components (background knowledge; local problem; intended aim; and who, what and why of intervention)	Describes 3/10 components (indicated main factors contributing to choice of intervention; study design for measuring its impact; explains how method was applied)	Describes 2/5 components (instruments to measure effectiveness of implementation, primary and secondary outcomes)	Describes 1/4 components (details of qualitative and quantitative methods)	Describes 2/4 components (documents degree of success in implementation; describes how and why the initial plan evolved)	Describes 4/5 components (presents data on changes observed in the care delivery process; presents data on changes observed in measures of patient outcome; considers benefits, harms, unexpected results, problems, failures; presents evidence regarding strength of association between intervention and changes)	Describes 3/5 components (summary, interpretations, conclusions)	19/38
2. King, Ben-Tovim, Bassham (2006)	Describes 3/5 components (local problem; intended aim; and who, what and why of intervention)	Describes 4/10 components (setting, intervention and components /parts; indicated main factors contributing to choice of intervention, implementation plan)	Describes 1/5 components (primary and secondary outcomes)	Describes 2/4 components (details of qualitative and quantitative methods; aligns unit of analysis with the intervention)	Describes 3/4 components (relevant elements of setting or settings; explains the actual course of the intervention; describes how and why the initial plan evolved)	Describes 3/5 components (presents data on changes observed in the care delivery process; presents data on changes observed in measures of patient outcome; presents evidence on strength of association between intervention and changes)	Describes 3/5 components (summary; limitations; conclusions)	19/38
3. Raab, Andrew-JaJa, Condel, et al.(2006)	Describes 3/5 components (background knowledge; intended aim; and who, what and why of intervention)	Describes 5/10 components (setting; intervention and components/ parts; indicated main factors contributing to choice of intervention; study design for measuring impact intervention; explains how method was applied)	Describes 1/5 components (methods used to assure data quality and adequacy)	Describes 3/4 components (details of qualitative and quantitative methods; specifies degree of expected variability; describes analytic method used to demonstrate effects of time)	Describes 2/4 components (explains the actual course of the intervention; documents degree of success in implementation)	Describes 2/5 components (considers benefits, harms, unexpected results, problems, failures; presents evidence regarding strength of association between intervention and changes)	Describes 3/5 components (relation to other evidence, limitations, interpretations)	19/38
4. Raab, et al. (2006)	Describes 3/5 components (background knowledge; intended aim; and who, what and why of intervention)	Describes 6/10 components (setting; intervention and components/ parts; indicated main factors contributing to choice of intervention; expected change mechanisms; study design for measuring impact intervention; explains how method was applied)	Describes 0/5 components	Describes 1/4 components (describes analytic method used to demonstrate effects of time)	Describes 0/4 components	Describes 1/5 components (presents evidence regarding strength of association between intervention and changes)	Describes 4/5 components (relation to other evidence; limitations; interpretations; conclusions)	15/38
5. Shannon, et al. (2006)	Describes 3/5 components (background knowledge; intended aim; and who, what and why of intervention)	Describes 4/10 components (ethical issues; setting; intervention and components/ parts; Implementation plan)	Describes 1/5 components (primary and secondary outcomes)	Describes 2/4 components (aligns unit of analysis with the intervention; describes analytic method used to demonstrate effects of time)	Describes 2/4 components (explains the actual course of the intervention; documents degree of success in implementation)	Describes 4/5 components (presents data on changes observed in the care delivery process; presents data on changes observed in measures of patient outcome; considers benefits, harms, unexpected results, problems, failures; presents evidence regarding strength of association between intervention and changes)	Describes 4/5 components (summary; relation to other evidence; limitations; interpretations)	20/38
6. Kelly, Bryant, Cox et al. (2007)	Describes 4/5 components (background knowledge; local problem; intended aim; and who, what and why of intervention)	Describes 5/10 components (setting; intervention and components/parts; implementation plan; study design for measuring impact intervention; explains how method was applied)	Describes 3/5 components (instruments to measure effectiveness of implementation; contribution of components of intervention to effectiveness; primary and secondary outcomes)	Describes 1/4 components (aligns unit of analysis with the intervention)	Describes 2/4 components (explains the actual course of the intervention; documents degree of success in implementation)	Describes 2/5 components (presents data on changes observed in care delivery process; includes summary of missing data)	Describes 5/5 components (summary; relation to other evidence; limitations; interpretations; conclusions)	22/38
7. Kim, et al. (2007)	Describes 4/5 components (background knowledge; local problem; intended aim; and who, what and why of intervention)	Describes 5/10 components (ethical issues; setting; intervention and components/ parts; indicated main factors contributing to choice of intervention; study design for measuring impact intervention; explains how method was applied; internal and external validity)	Describes 2/5 components (instruments to measure effectiveness of implementation; contribution of components of intervention to effectiveness)	Describes 1/4 components (aligns unit of analysis with the intervention)	Describes 2/4 components (explains the actual course of the intervention; documents degree of success in implementation)	Describes 2/5 components (presents data on changes observed in care delivery process; includes summary of missing data)	Describes 4/5 components (summary; relation to other evidence; limitations; interpretations)	20/38
8. Raab, Grzybicki, Condel, et al. (2007)	Describes 3/5 components (background knowledge; intended aim; and who, what and why of intervention)	Describes 6/10 components (setting; intervention and components/parts; indicated main factors contributing to choice of intervention; implementation plan; study design for measuring impact intervention; explains how method was applied)	Describes 1/5 components (instruments to measure effectiveness of implementation)	Describes 1/4 components (describes analytic method used to demonstrate effects of time)	Describes 1/4 components (documents degree of success in implementation)	Describes 2/5 components (presents data on changes observed in care delivery process; considers benefits, harms, unexpected results, problems, failures)	Describes 3/5 components (summary; limitations; interpretations)	17/38
9. Shendell-Falik, Feinson, Mohr (2007)	Describes 4/5 components (background knowledge,; local problem; intended aim; and who, what and why of intervention)	Describes 4/10 components (setting; intervention; components/parts; indicated main factors contributing to choice of intervention; expected change mechanisms)	Describes 3/5 components (instruments to measure effectiveness of implementation; contribution of components of intervention to effectiveness; primary and secondary outcomes)	Describes 0/4 components	Describes 4/4 components (relevant elements of setting or settings; explains the actual course of the intervention; documents degree of success in implementation; describes how and why the initial plan evolved)	Describes 3/5 components (presents data on changes observed in the care delivery process; presents data on changes observed in measures of patient outcome; presents evidence regarding strength of association between intervention and changes)	Describes 2/5 components (summary; conclusions)	20/38
10. Wood, Brennan, Chaudhry, et al. (2008)	Describes 3/5 components (background knowledge; intended aim; and who, what and why of intervention)	Describes 2/10 components (setting; intervention and components/parts)	Describes 1/5 components (primary and secondary outcomes)	Describes 0/4 components	Describes 1/4 components (actual course of the intervention)	Describes 1/5 components (evidence regarding strength of association between intervention and changes)	Describes 3/5 components (summary; relation to other evidence; conclusions)	11/38
11. Reid, et al. (2009)	Describes 4/5 components (background knowledge; local problem; intended aim; and who, what and why of intervention)	Describes 7/10 components (ethical issues; setting; intervention and components/parts; indicated main factors contributing to choice of intervention; study design for measuring impact of intervention; explains how method was applied; internal and external validity)	Describes 3/5 components (instruments to measure effectiveness of implementation; primary and secondary outcomes; methods used to assure data quality and adequacy)	Describes 3/4 components (details of qualitative and quantitative methods; aligns unit of analysis with the intervention; describes analytic method used to demonstrate effects of time)	Describes 0/4 components	Describes 3/5 components (presents data on changes observed in measures of patient outcome; presents evidence regarding strength of association between intervention and changes; includes summary of missing data)	Describes 5/5 components (summary; relation to other evidence; limitations; interpretations; conclusions)	25/38
12. Auerbach, et al. (2010)	Describes 3/5 components (background knowledge; intended aim; and who, what and why of intervention)	Describes 8/10 components (setting; intervention and components/parts; indicated main factors contributing to choice of intervention; implementation plan; plan for assessment of implementation; study design for measuring impact of intervention; explains how method was applied; internal and external validity)	Describes 2/5 components (instruments to measure effectiveness of implementation; primary and secondary outcomes)	Describes 3/4 components (details of qualitative and quantitative methods; aligns unit of analysis with the intervention; describes analytic method used to demonstrate effects of time)	Describes 2/4 components (relevant elements of setting or settings; documents degree of success in implementation)	Describes 5/5 components (presents data on changes observed in the care delivery process; presents data on changes observed in measures of patient outcome; considers benefits, harms, unexpected results, problems, failures; presents evidence regarding strength of association between intervention and changes; includes summary of missing data)	Describes 4/5 components (summary; relation to other evidence; limitations; interpretations)	27/38
13. Ravikumar, et al. (2010)	Describes 3/5 components (background knowledge; intended aim; and who, what and why of intervention)	Describes 7/10 components (setting; intervention and components/parts; indicated main factors contributing to choice of intervention; implementation plan; study design for measuring impact of intervention; explains how method was applied; internal and external validity)	Describes 1/5 components (primary and secondary outcomes)	Describes 3/4 components (details of qualitative and quantitative methods; aligns unit of analysis with the intervention; describes analytic method used to demonstrate effects of time)	Describes 4/4 components (relevant elements of setting or settings; explains the actual course of the intervention; documents degree of success in implementation; describes how and why the initial plan evolved)	Describes 3/5 components (presents data on changes observed in measures of patient outcome; considers benefits, harms, unexpected results, problems, failures; presents evidence regarding strength of association between intervention and changes)	Describes 4/5 components (relation to other evidence; limitations; interpretations; conclusions)	25/38
14. Hwang, Lee, Shin (2011)	Describes 4/5 components (background knowledge; local problem; intended aim; and who, what and why of intervention)	Describes 4/10 components, (setting; intervention and components parts; indicated main factors contributing to choice of intervention; study design for measuring intervention)	Describes 2/5 components (primary and secondary outcomes; methods used to assure data quality and adequacy)	Describes 0/4 components	Describes 0/4 components	Describes 3/5 components (data on changes observed in the care delivery process; data on changes observed in measures of patient outcome; considers benefits, harms, unexpected results, problems, failures)	Describes 5/5 components (summary; relation to other evidence; limitations; interpretations; conclusions)	18/38
15. Collar, et al. (2012)	Describes 1/5 components (intended aim)	Describes 6/10 components (intervention and components/parts; indicated main factors contributing to choice of intervention; implementation plan; study design for measuring impact of intervention; explains how method was applied; internal and external validity)	Describes 2/5 components (primary and secondary outcomes; reports validity and reliability of instruments)	Describes 2/4 components (details of qualitative and quantitative methods; aligns unit of analysis with the intervention)	Describes 0/5 components	Describes 4/5 components (presents data on changes observed in the care delivery process; presents data on changes observed in measures of patient outcome; considers benefits, harms, unexpected results, problems, failures; presents evidence regarding strength of association between intervention and changes)	Describes 4/5 components (relation to other evidence; limitations; interpretations; conclusions)	19/38
16. Krening, Rehling-Anthony, Garko (2012)	Describes 4/5 components (background knowledge; local problem; intended aim; and who, what and why of intervention)	Describes 5/10 components (setting; intervention and components/parts; indicated main factors contributing to choice of intervention; implementation plan; expected change mechanisms)	Describes 3/5 components (instruments to measure effectiveness of implementation; primary and secondary outcomes; explains methods used to assure data quality and adequacy)	Describes 0/4 components	Describes 4/4 components (relevant elements of setting or settings; explains the actual course of the intervention; documents degree of success in implementation; describes how and why the initial plan evolved)	Describes 4/5 components (presents data on changes observed in the care delivery process; presents data on changes observed in measures of patient outcome; considers benefits, harms, unexpected results, problems, failures; presents evidence regarding strength of association between intervention and changes)	Describes 4/5 components summary; limitations; interpretations; conclusions)	20/38
17. Murray, Christen, Marsh, et al.(2012)	Describes 4/5 components (background knowledge; local problem; intended aim; and who, what and why of intervention)	Describes 6/10 components (setting; intervention and components/parts; indicated main factors contributing to choice of intervention; implementation plan; expected change mechanisms; internal and external validity)	Describes 3/5 components (instruments to measure effectiveness of implementation; primary and secondary outcomes; methods used to assure data quality and adequacy)	Describes 2/4 components (details of qualitative and quantitative methods; aligns unit of analysis with the intervention)	Describes 2/4 components (relevant elements of setting or settings; describes how and why the initial plan evolved)	Describes 4/5 components (presents data on changes observed in the care delivery process; presents data on changes observed in measures of patient outcome; presents evidence regarding strength of association between intervention and changes; includes summary of missing data)	Describes 5/5 components (summary; relation to other evidence; limitations; interpretations; conclusions)	23/38
18. Liss, et al. (2013)	Describes 4/5 components (background knowledge; local problem; intended aim; and who, what and why of intervention)	Describes 4/10 components, (setting; indicated main factors contributing to choice of intervention; study design for measuring intervention; internal and external validity)	Describes 3/5 components (primary and secondary outcomes; validity and reliability of instruments; explains methods used to assure data quality and adequacy)	Describes 1/4 components (details of qualitative and quantitative methods)	Describes 1/4 components (characterizes relevant elements of setting or settings)	Describes 2/5 components (presents data on changes observed in measures of patient outcome; presents evidence regarding strength of association between intervention and changes)	Describes 5/5 components (summary; relation to other evidence; limitations; interpretations; conclusions)	20/38

### Types of redesign interventions

Table 3 summarizes the redesign interventions and study methods used. The objective of most studies was the implementation and evaluation of a specific redesign intervention. Improving quality of care was explicitly stated as an objective in seven studies [12, 15, 18, 20, 23, 25, 26]. Half of the redesign interventions implemented the approach known as lean thinking/Toyota production system (N = 9) [12, 14, 15, 21, 24–28]. Two studies described the implementation of the concept of patient-centered medical home [17, 20], and three described more general forms of process redesign (structure redesign vs. process redesign [23], evidence-based redesign [18], nurse practitioner-led practice redesign [29]). Other interventions included a general process improvement project [16], appreciative inquiry [22], a hospitalist-led co-management neurosurgery service [13] and a continuum of care [19].

**Table 3:** Overview of types of redesign interventions and methods used in included studies

Reference (author names, publication year, country)	Intervention		Methods			
	Objectives	Type of intervention	Study design	Unit of analysis (project sample size), study sample size	Intervention components	Length of follow-up
1. Pennell, et al. (2005) USA	To produce substantiated practice changes in glycemic management and improved outcomes for coronary artery bypass surgery patients	NP-led practice redesign	Before-and-after study	N = 103 (Before group = 41; After group = 62).	1. New cardiothoracic team established, including advanced practice nurses; 2. 2. Implementation of new tools and guidelines	Not mentioned
2. King, Ben-Tovim, Bassham (2006) Australia	Streamlining patient care at the ED to reduce overcrowding	Lean thinking	Before-and-after study	Before: N = 49075 presentations to the ED; After: N = 50337 presentations to the ED.	1. Process mapping (incl. value stream map); 2. Restructuring of patient flow; streamlining in relation to predicted outcome	12 months
3. Raab, Andrew-JaJA, Condel, et al. (2006) USA	Improving Papanicolaou test quality and reducing medical errors by using Toyota production system methods	Toyota production system	Non-concurrent cohort study with control-group and comparison of retrospective consecutive case data from previous year for same time frame	Women with ASC US (atypical squamous cells of undetermined significance) diagnosis	1. Choosing a target for improvement; 2. Problem Analysis; 3. Intervention design; 4. Pretest; 5. Implementation; 6. Evaluation	Not mentioned
4. Raab, et al. (2006) USA	Determine whether the Toyota production system process redesign resulted in diagnostic error reduction for patients who underwent cytologic evaluation of thyroid nodules	Toyota production system	Longitudinal before-and-after, non-concurrent cohort study	2,424 patients with thyroid gland nodule	1. Development and use of a standardized diagnostic terminology scheme; 2. Expansion of an immediate interpretation service	Not mentioned
5. Shannon, et al. (2006) USA	Eliminating central line-associated bloodstream (CLAB) infections in ICUs by employing the principles of Toyota production system adapted to health care	(Lean thinking) Toyota production system	Before-and-after study	49 patients with CLAB admitted to medical intensive care unit and coronary care unit between July 2002 and June 2003. 10 residents, 10 fellows, 8 attending physicians, 16 nurses, 6 nurse aides and 5 personnel	Real-time problem-solving with help of the Toyota production system	34 months
6. Kelly, Bryant, Cox, et al. (2007) Australia	Analyze ED patient flow processes using task analysis and lean thinking; re-engineer these processes to improve flow through the ED for all groups of patients	Lean thinking	Before-and-after study	31570 patients admitted to emergency department	Choosing a target for improvement; problem analysis; intervention design; pretest; implementation; and evaluation	Not mentioned
7. Kim, et al. (2007) USA	Implement a lean project to improve patient care access and reduce excess work in providing palliative radiation therapy to patients referred for bone or brain metastases	Lean thinking	Before-and-after study	1600 patients in total/year, 15 % have bone or brain metastases	Applied the principles and tools of lean thinking	Not mentioned
8. Raab, Grzybicki, Condel, et al. (2007) USA	To measure the effect of implementation of a lean quality improvement process on the efficiency and quality of a histopathology lab section	Lean thinking	Non-concurrent interventional cohort study with control group and pre-post measurement	One histopathology section of anatomical pathology laboratory	1. Education of staff; 2. Determining current condition; 3. Designing and implementing multiple (200) interventions; 4. Sustaining the “perfecting patient care” learning line	Not mentioned
9. Shendel-Falik, Feinson, Mohr (2007) USA	Develop and implement a standardized approach to “hand-off” communications, including an opportunity to ask and respond to questions	Appreciative inquiry	Before-and-after study	Patients being transitioned from the ED to the telemetry unit and the associated care providers involved in the handoff	A 5D cycle of appreciative inquiry (definition, discover, dream, design, destiny) with 5 improvement projects: 1. A welcome script,; 2. Safety assessments; 3. Standardized transfer report; 4. Low-risk cardiac transport protocol; 5. Interpersonal relationships	6 months
10. Wood, Brennan, Chaudhry, et al. (2008) USA	To improve the quality and safety of patient care and to improve the efficiency and satisfaction of all team members, including physicians	Lean thinking	Before-and-after study	1157 consecutive clinical notes before and 257 clinical notes after implementation; 137 physicians and 12 allied health staff members	Standardized process of patient care that included collaborative work between physicians and appropriately trained clinical assistants; the rooming process	Not mentioned
11. Reid, et al. (2009) USA	1. Maintain or enhance patient care experiences; 2. Reduce physician and care team burnout; 3. Improve clinical quality scores; 4. Reduce emergency, specialty and avoidable hospitalization use and costs	Patient-centered medical home	Before-and-after study	One intervention clinic and 19 control clinics; 8094 patients were included at the PCMH clinic and 228,510 patients were included at the control clinics	1. Structural changes; 2. Point-of-care process changes; 3. Patient outreach changes; 4. Management process changes	12 months
12. Auerbach, et al. (2010) USA	The co-management neurosurgery service (CNS) was implemented in response to changes in care—primarily reducing availability of physicians for ward patients—which resulted from resident duty hour reductions	Hospitalist-led co-management neurosurgery service (CNS)	Before-and-after study with control group	A total of 7596 patients were admitted to the neurosurgery service during the study period: 4203 (55.3 %) before July 1, 2007, and 3393 (44.7 %) after CNS implementation	Co-management: shared management of surgical patients between surgeons and hospitalists	18 months
13. Ravikumar, et al. (2010) USA	Reduce mortality by enhancing continuity and co-management throughout hospital stay; minimize errors at transition points; increase throughput; reduce length of stay	Continuum of care	Before-and-after study with control group	Pilot study: one intervention and one control hospital. Validation study: one hospital department as intervention group and the entire hospital as control cohort CoC study: one hospital	1. Surgical Continuum of Care (SCoC); 2. Continuum of Care (CoC)	Pilot study: 3 years; Validation study: 3 years; CoC study: 6 months
14. Hwang, Lee, Shin (2011) South Korea	To shorten processing time and improve service quality	Structure redesign vs. process redesign	Before-and-after study	Two teaching hospitals. At Guro hospital (layout redesign) the final sample sizes were 291 patients at baseline and 170 patients at follow-up. At Anam hospital (critical pathway implementation) the final sample sizes were 273 patients at baseline and 125 patients at follow-up	1. Structure-oriented approach: improvement of the physical structure of the ER operations by remodeling the hospital’s layout; 2. Process-oriented approach: implementation of critical pathways and protocols	12 months
15. Collar, et al. (2012) USA	To determine whether systematic implementation of lean thinking in an academic otolaryngology operating room improves efficiency and profitability and preserves team morale and educational opportunities; all staff working at one surgeon’s operating room	Lean thinking	Before-and-after study (18-month prospective quasi-experimental study)	144 cases were included in the baseline period and 55 cases in the intervention period (follow-up)	1. Visualization of the current state of the perioperative work process in the form of a swim lane diagram; 2. Identification of waste; 3. Root cause analysis for key waste items; 4. Creation of new swim lanes and a standard work matrix	6 months
16. Krening, Rehling-Anthony, Garko (2012) USA	To decrease risk exposure in the use of oxytocin administration hospitals of Centura Health	A process improvement project; standardized evidence-based protocol and processes across the healthcare system	Before-and-after study	Nine hospitals of Centura Health, delivering obstetric care	1. A standardized oxytocin mixture; 2. Low-dose administration guidelines; 3. Utilization of safety checklists to assure fetal and maternal well-being before initiation of oxytocin and increases in oxytocin dosages; 4. A standardized order set; 5. An educational handout for pregnant woman on oxytocin usage	12 months
17. Murray, Christen, Marsh, et al. (2012) Scotland	Redesign of the new-patient fracture clinic, with the objective of: improving patient care, trainee education, interprofessional relations and clinic efficiency	Evidence-based redesign	Not mentioned	301 consecutive patients attending the new-patient fracture clinic over a 3-week period in the summer of 2010, compared to 346 consecutive patients during a 3-week period exactly one year previously. Adequate data available for 240 patients (80 %) in 2010 and 296 patients (86 %) in 2009	1. Investigate existing conditions before introducing the new clinic model; 2. identify problems and delineate potential improvements; 3. Redesigned the new-patient fracture clinic; 4. Implemented change; 5. Documented outcomes	3 months
18. Liss, et al. (2013) USA	Providing patients with a continuous source of whole-person primary care; increasing patient access and satisfaction with care and reducing total costs	Patient-centered medical home	Controlled before-and-after study	One Group Health clinic as intervention site and 19 Group Health Clinics as controls. The final study population included 37,930 adults with diabetes, hypertension and/or CHD, with 1181 patients paneled to the PCMH prototype clinic and 36,757 patients paneled to other clinics	1. Increased primary care staffing; 2. Physicians paired in dyads with medical assistants; 3. Standard in-person primary care office visits lengthened to 30 min; 4. Virtual medicine contacts; 5. Rerouting patients’ calls; 6. Creation of collaborative care plans; 7. Provider outreach to manage monitoring tests	21 months

Fourteen studies were performed in the USA [12, 13, 15–17, 19–22, 25–29], two in Australia [14, 24], one in South Korea [23] and one in Scotland [18]. Most took place in a hospital setting (N = 12) [13–16, 19, 21–24, 27–29]; others were conducted in primary care (N = 3) [12, 17, 20], a specialized clinic (N = 1) [18] or a laboratory (N = 2) [25, 26]. Length of follow-up ranged from three [18] to 48 [27] months with a median of 12 months, though five studies did not mention its duration [12, 14, 15, 26, 29]. Patients were the most common unit of analysis (N = 14) [13–15, 17, 18, 20–25, 27–29]. However, some studies reported on staff (N = 2) [12, 21] or clinical notes (N = 1) [12] while a few did not define the unit of analysis (N = 3) [16, 19, 26]. Mean sample size was 27,932.87(SD = 61,506.98), ranging from 49 [21] to 228,510 [20]. Thirteen studies used a before-and-after design (N = 12) [12, 14–16, 20–24, 27–29], while five used a controlled before-and-after design [13, 17, 19, 25, 26].

In summary, half of the redesign interventions were characterized as ‘lean thinking’ and took place in a hospital setting. Length of follow-up and sample size diverged widely, and most studies used an uncontrolled before-and-after design to evaluate the effectiveness of the intervention.

### Effects of redesign on quality of care

Table 4 summarizes the outcomes of the studies. All reported improvements as a result of process redesign, while three [14, 20, 23] also found declines in quality. Significant improvements were mentioned in 15 studies [13, 14, 16–21, 23–28], mostly gains in effectiveness [16–21, 25, 27] and/or efficiency [14, 17–20, 23, 24, 26, 28]. Outcome measures showed great variance between studies. However, ‘effectiveness’ and ‘efficiency’ were discussed most (11 studies reported on both dimensions [13, 14, 16–22, 25, 29]). Changes in efficiency were demonstrated by 17 studies [12–25, 28, 29]. Efficiency was improved by decreasing hospitalization rates [17, 20], process times (including time to treatment) [14, 23, 24, 28], length of hospital stay [19, 23, 29]; by a shift in the writing of clinical notes [12], savings on (estimated) costs [13, 16, 19, 20, 25, 28], raising provider productivity [21, 22, 26] and reducing process steps and variability [15, 18, 24, 25]. Efficiency also deteriorated: an increase was shown in process time for a sub-category of patients [14, 23], in specialty care visits [20] and in specialty care costs [20].

**Table 4:** Overview of outcomes of redesign interventions in included studies

Study reference (author names, publication year)	Quality of care						Other outcomes
	Effectiveness	Efficiency	Timeliness	Patient-centeredness	Safety	Equity of care	
1. Pennell, et al. (2005)	- Improved basal diabetes medications being ordered prior to discontinuing the IV insulin infusion (0 % → 76.9 %) - Use of sliding scale insulin increased in undiagnosed patients (16 % → 21 %) - Use of basal medications while on sliding scale insulin improved for diagnosed patients (56.3 % → 69 %) - Increased number of documented blood glucose tests ordered for undiagnosed patients (2.8/day → 4.3/day) - Improved diabetic patients’ blood glucose test values (88 % → 71 % range 140 to 299 mm/dL)	- The Average Length Of Stay (ALOS) for the overall population was reduced by 1.2 days - The ALOS for diagnosed patients increased by 2.6 days - The ALOS for undiagnosed patients decreased by 4.6 days - The ALOS for diagnosed patients for the year was shorter than for undiagnosed patients - Patients with a primary diagnosis of coronary artery bypass with cardiac cath with complications had a significantly longer ALOS at 12.9 days - The ALOS of undiagnosed patients with coronary bypass with cardiac cath dropped after implementation	n/a	n/a	- Percentage of undiagnosed patients with postoperative infection dropped (16 % → 9.1 %) - Percentage of diagnosed patients with a postoperative infection increased (0 % → 10 %) - Diagnosed patients had fewer postoperative infections than undiagnosed patients (6.7 % vs. 12 %)	n/a	n/a
2. King, Ben-Tovim, Bassham (2006)	n/a	- Flattening of the review times - Marked reduction in the variability of time spent waiting for review - Time to initiation of meaningful treatment significantly decreased - Time to see a doctor decreased - A slight increase in overall compliance to meeting triage waiting times - Percentage of all patients attending but not waiting to be seen after initial triaging fell significantly - Decrease in patients presenting to the ED and waiting for more than 8 h before being admitted or discharged - Significant decrease in mean time spent in the ED - Significant decrease in time spent in the ED of patients being admitted - Significant decrease in time spent in the ED of patients being discharged - Decrease of overall time spent in the department - - Decrease of time spent in the department before discharge	n/a	n/a	- No incidents of concerns associated with practice change - Overall sense of a greater degree of patient safety, and sense of control among staff	n/a	n/a
3. Raab, Andrew-JaJA, Condel, et al. (2006)	- Significant decrease of Papanicolaou tests lacking a transformation zone component (9.9 % → 4.7 %)	- Reduced number of equivocal Papanicolaou test diagnoses (7.8 % → 3.9 %) - Decreased costs - Less additional testing (76 % → 29.4 %) - Decreased laboratory - time and effort in the screening of slides	n/a	n/a	- More women being diagnosed with appropriate categories - - Decrease of error frequency per correlating cytologic-histologic specimen pair (9.52 % → 7.84 %)	n/a	n/a
4. Raab, et al. (2006)	- Improvement: - Significantly higher diagnostic accuracy (70.2 % → 90.6 %). - Decrease of Fine Needle Aspiration (FNA) (1543 → 1176 cases) - Significant decrease in repeated FNA rate (12.7 % → 7.7 %) - Significant decrease in non-interpretable rate for immediate interpretation service (23.8 % → 7.8 %) - Deteriorations: - Significant increase in non-interpretable rate (5.8 → 19.8 %) at terminology standardization	n/a	n/a	n/a	- Significantly fewer false-negative diagnoses (4.8 % → 19.1 %) - Significantly fewer patients had surgery (23.6 % → 19.9 %) - Deteriorations: - - No significant increase in false-positive rate (22.6 → 26.3 %)	n/a	n/a
5. Shannon, et al. (2006)	- -Significant increase in line days (4,687 days → 7,716 days)	- Increase in admissions (11 %) - Increased acuity - Near doubling of line use without adding new staff or more beds - - Reduced need to compensate for ineffective processes	n/a	n/a	- Reduced line infection rates after intervention (10.5/1000 → 0.39/1000 line days) - - Significantly reduced line infection associated mortalities (51 % → 0 %)	n/a	- More time to be involved in direct patient care - - More time for staff to solve problems
6. Kelly, Bryant, Cox, et al. (2007)	- Increased and sustained proportion of discharged patients (92 %)	- Improvements: - Significant reduction of overall total ED department time (12 min) - Significant reduction of total ED time for triage category 4 and 5 patients (14 and 18 min respectively) - Deteriorations: - Significant (*) increase in total ED time for category 1, 2 and 3 patients (9, 13 and 7* minutes respectively)	Significant reduction in waiting time, overall and in triage categories 2–5 (3, 2, 5, 7 and 11 min respectively) Increased bed requests within target time (73 %)	n/a	- Episodes of ambulance bypass significantly decreased (120 → 54)	n/a	- - 90 % of staff reported that they believed the ED ran better after the change
7. Kim, et al. (2007)	n/a	- Reduction of process steps (16) to treatment - Decrease of variability	Increase of percentage of new patients with brain or bone metastases receiving consultation, simulation, and treatment on the same day (43 % → 94 %) - Process time remained stable (225 min) while wait time decreased (1 week → 1 day)	n/a	- Fewer process errors in routing patient to appointment times	n/a	- n/a
8. Raab, Grzybicki, Condel, et al. (2007)	n/a	- Significantly increased productivity (3439 to 4047 work units/FTE) - Decrease of expenditure - Decreased specimen Turn Around Time (TAT) (9.7 h → 9.0 h)	n/a	n/a	n/a	n/a	n/a
9. Shendel-Falik, Feinson, Mohr (2007)	- Nutritional assessment improved by 11 % - Completion of skin assessment in the ED improved by 70 % - - Compliance with cardiac enzyme regimen improved by 9.2 %	- Percentage of telemetry patients able to be transported without a cardiac monitor increased by 60 % - 67.5 h of nursing time per month were saved.	n/a	- Overall patient satisfaction improved on nursing issues (10.2 %) - Satisfaction with personal issues improved (9 %) - ED rating improved (23.3 %)	n/a	n/a	- - Improved nurse satisfaction and teamwork
10. Wood, Brennan, Chaudhry, et al. (2008)	n/a	- Shift from clinical notes dictated by physicians to clinical notes written by clinical assistants - 21 % of the note was authored by clinical assistants and 79 % by physicians	n/a	n/a	- Significant improvements: - Increased physician identification (from 57 % to 88 %) - Increased allergy documentation (from 52 % to 70 %) - Increased advance directives documentation (from 2 % to 83 %) - Improved medication list completeness (from 32 % to 91 %)	n/a	- - Improved physician satisfaction
11. Reid, et al. (2009)	- PCMH patients had significantly better performance on - each of the composite measures compared with 19 other clinics at baseline - Significant improvement of composite quality of care at the PCMH compared to baseline (4 %) and control groups (1.4 %)	- Improvements - PCMH patients received fewer in-person primary care visits (6 %) - PCMH patients had significantly fewer ED visits (29 %) - PCMH patients had significantly fewer hospitalizations for ambulatory care-sensitive conditions (11 %) - PCMH patients had lower ED costs ($54 per patient per year) - Deteriorations: - PCMH patients had significantly more specialty care visits (8 %) - PCMH patients had higher primary care costs per patient per year ($16 per patient per year) - PCMH patients had higher specialty care costs ($37 per patient per year)	n/a	- PCMH patients reported significantly better experience with their care - PCMH patients reported significantly higher scores on quality of doctor-patient interactions, coordination of care, patient activation/involvement and goal setting/tailoring - Patients in the control groups reported significantly higher scores for patient activation/involvement and goal setting/tailoring. - Patients at the PCMH clinic reported significantly higher scores on quality of doctor-patient interaction, shared decision making, coordination of care, access, patient activation/involvement and goal setting/tailoring	n/a	n/a	- Emotional exhaustion among physicians and physician assistants was reported significantly less frequently (20 %) at the PCMH clinic
12. Auerbach, et al. (2010)	- No significant differences in mortality rate - No significant differences in readmission after 30 days	- Moderate decrease in adjusted hospital cost equivalent to a savings of $1439 per admission	n/a	- Statistically significant increases in the odds for a higher score in the co-management cohort for 3 questions: degree to which staff responded to concerns; cheerfulness of the hospital; and degree to which staff addressed patients’ emotional needs. - - No significant differences in overall rating of the hospital experience and likelihood of recommending the hospital	n/a	n/a	- Non-nursing professionals support CNS; significantly improved attention to medical issues during hospitalization and at discharge - - Nursing perceptions of the CNS’s effect on patient care were uniformly positive, with strongest positive change again being seen on questions regarding treatment of medical issues during hospitalization
12. Ravikumar, et al. (2010)	- - Significant improvement of readmission rates	- Significant reduction of total hospital patient days for patients being discharged from SICU to the regular beds or to PCU - Net cost savings - Decreased SICU Length Of Stay (LOS) - Decreased PCU LOS: - Decreased total hospital LOS SICU - Decreased total hospital LOS PCU - Cost savings: $851,511 to $2,007,388 per year. - For DRG 148, reduction of variable cost was $452,000 per year	n/a	n/a	- Overall surgical mortality significantly decreased, with a corresponding improvement in mortality index for surgical DRGs	n/a	n/a
13. Hwang, Lee, Shin (2011)	n/a	- Improvement hospital layout remodeling: - Significant (*) decrease of the mean time for the five processes: registration (7.78 %); CT/MRI enrollment (8.75 %); Complete Blood Count (CBC) sample collection (5.98 %); Prothrombin Time (PT)/Partial Thromboplastin Time (PTT) sample collection (19.73 %*); and CBC report (21.63 %*) - Time reduction in PT/PTT sample collection process - Significant reduction of total time from arrival to treatment (10.37 %) - Significant decrease in length of stay (from 10.02 to 8.6 days) - Significantly lower hospital charges (10.25 %) - Deterioration hospital layout remodeling: - Significant increase of CT/MRI and PT/PTT reporting process time (from 29.6 to 64.81 min; 28.99 %*) - Improvement process redesign: - Significant (*) decrease in process times: registration (22.76 %); CT/MRI enrollment (18.29 %); CBC sample collection (10.28 %); PT/PTT sample collection (14.32 %*); CT/MRI scan report (15.71 %*); PT/PTT report (3.59 %) - Significant decrease in time from arrival to treatment (15.75 %) - Significant decrease in LOS (from 12.98 to 9.25 days) - Significantly lower hospital charges (16 %) - Deterioration process redesign: - - Increase in CBC report time (67.96 %)	n/a	n/a	n/a	n/a	n/a
14. Collar, et al. (2012)	n/a	- No significant difference in case length - Mean Turn Over Time (TOT) was significantly shortened - Turn Around Time (TAT) was significantly shortened - Percentage of TOTs of 30 min increased - Percentage of TATs of 60 min increased - Approximately 4,500 min of added capacity yielded - - Annual opportunity revenue for a single OR used twice weekly is approximately $330,000	n/a	n/a	n/a	n/a	- Significantly improved team morale - - Operating Room Environment Measure did not change significantly
15. Krening, Rehling-Anthony, Garko (2012)	- Decrease in average length of labor on oxytocin for both primigravidas (10 h → 9.5 h) and multigravidas (8 h → 6.5 h). - Significant decrease in hours receiving oxytocin for both primigravidas (9.9 h → 8.78 h) and multigravidas (7.8 h → 6.22 h) - Decrease in primary cesarean rate (61 % → 56 %)	- A theoretical saving of at least $173,000 per year if volume remains constant, caused by reduced labor length - A theoretical saving of approximately $286,000 per year, caused by reduced primary cesareans	n/a	n/a	- Significant decrease in overall incidence of tachysystole (54 % → 20 %)	n/a	n/a
16. Murray, Christen, Marsh, et al. (2012)	- Significant decrease in overall ‘return rates’ (162 → 97 patients) - Discharge rate improved (22 % → 25 %)	- Significant decrease in proportion of patients requiring additional physical review by a consultant (89 → 22 patients) - Significant improvement in utilization of the nurse-led fracture clinic (38 → 55 referrals)	n/a	n/a	- Significant increase in proportion of cases receiving primary consultant input (98 → 202 patients)	n/a	- Significant improvements in median scores of staff perception of education, provision of senior support, morale and overall perception of patient care. - ER staff said the new style clinic was educational, practice-changing and improved interdisciplinary relations - - Reduction of official incidence rates IR1 reports
17. Liss, et al. (2013)	- Significantly improved disease conditions for patients with diabetes; 4 % more likely to have A1C under 9.0 %, mean A1C 0.20 % lower - Significant improved follow-up and disease conditions for patients with CHD; 11 % more likely to have LDL below 100 mg/dL at follow-up, mean LDL was 2.20 mg/dL lower - Improved disease conditions for patients with hypertension; 5 % more likely to have blood pressure below 140/90 mmHg, not significant	- Significant decrease (23 %) in ambulatory care sensitive hospitalizations for patients at the PCMH - Significant decrease (4 %) in inpatient admissions for patients at the PCMH - Significant decrease (18 %) in ED and urgent care contacts	n/a	n/a	n/a	n/a	n/a

Changes in effectiveness were demonstrated in 12 studies [13, 14, 16–22, 25, 27, 29]. These reported improvements in disease conditions [17, 20, 29] and adequate treatment usage [16, 22, 29] as well as increases in discharged patients [14, 18] and diagnostic accuracy [25, 27].

Two studies [14, 15] found changes in timeliness as a result of process redesign, which reduced waiting time. Changes in patient-centeredness were demonstrated in three studies [13, 20, 22]: improvements in patient satisfaction or experiences [13, 20, 22]; higher scores on doctor-patient interaction; and better coordination of care [20]. Changes in patient safety were found in 11 studies [12, 14–16, 18, 19, 21, 24, 25, 27, 29]: increased physician identification [12]; improved documentation [12]; a decrease in complications [14, 16, 19, 21, 29]; fewer errors in routing patients to appointments [15]; fewer false-negative diagnoses [25, 27]; and an overall sense of improvement in patient safety [24].

None of the studies measured equity of care. Eight mentioned other outcomes unrelated to the six quality dimensions, such as changes in provider satisfaction [12, 22], staff perceptions of the implemented change [13, 14, 18, 21], changes in team morale [28], or changes in incident rates [18].

## Discussion

The need to redesign healthcare processes in order to address deficits in quality of care and create more sustainable care processes is acknowledged worldwide [2, 3, 5]. The effects of process redesign have not been clearly described, however [5, 6]. By synthesizing evidence from 18 studies in the international literature, this systematic review contributes to a better understanding of the influence of process redesign interventions on quality of care. It suggests that they have positive effects on certain aspects of quality. However, the full impact cannot be determined on the basis of the literature. Studies differed in the type of redesign implemented, study setting, methods used for evaluation, and outcome measures. All types of intervention seemed to improve outcomes in one or more respects. Nonetheless, it is not clear which type of redesign has the most potential in a particular setting. Efficiency, effectiveness and patient safety gains were best described in the included studies, while the effects on patient-centeredness, timeliness and equity of care received little attention.

Applying the SQUIRE guidelines demonstrated that overall the reporting was weak. Given the study designs, the results are subject to bias, as changes in the research settings might be responsible for the effects [30, 31]. In addition, changes in process might have been induced by background factors [31]. Longitudinal effects of redesign interventions were hardly evaluated, as follow-up varied from three to 48 months with a median of 12 months. The methodological problems of studies reporting on quality improvement interventions like process redesign are well known [6, 31–34]. Yet the methodology of the studies covered here was no better than in preceding studies. These weaknesses form potential threats to the internal and external validity of the findings. Unless a more uniform and robust evaluation of process redesign interventions is carried out, general conclusions cannot be drawn about their impact on quality of care.

To the best of our knowledge, this is the first systematic review of the effect of process redesign on quality of care, using broad definitions for both study setting and types of redesign. Elkhuizen et al. [6] performed a systematic review of the evidence of business process redesign in hospital settings until 2004. However, that review included studies combining multiple interventions, which made comparison impossible. Those authors concluded that studies were hard to find and lacked a clear and consistent research methodology. In that light, they recommended the development of reporting guidelines.

Specific redesign interventions have been reviewed recently. In one, Mazzocato et al. [35] reviewed the ‘lean-thinking’ literature from a realist perspective, focusing on the mechanisms through which ‘lean thinking’ operated. The authors identified positive effects of lean implementation in all included studies and common contextual factors interacting with components of the lean interventions that triggered the change mechanisms. Here too, the use of unclear study designs or outcome measures is mentioned. The authors suspect publication bias, as only positive effects were being reported.

The impact of quality-improvement collaboratives was reviewed by Schouten et al. [36]. Although the outcomes were positive, the strength of evidence was limited by methodological constraints due to weak study designs, and the authors suspect positively biased findings. Implementation of the concept ‘patient-centered medical home’ was reviewed by Jackson et al. [37], who showed small positive effects on patient experience and care delivery. There too, the strength of evidence was moderate to low. Publications were hard to find, evidence was fragmented, and comparison between studies was hard if not impossible.

The findings of the present review are therefore in line with those of earlier studies on this topic in the sense that a broad perspective on redesign interventions and settings generates similar results.

### Limitations

Even though a systematic approach guided this review, the findings might be subject to some bias, which should be kept in mind when interpreting them.

First, publication bias might be present: most of the studies report on positive findings, and there is a general tendency in scientific literature to over-represent positive results [38]. As previous research on this topic also raised concerns about publication bias, this issue is pertinent to this review too. It is unlikely that using predefined redesign concepts would have addressed this problem, as publication bias was a concern in reviews that did use such concepts [35], underlining the need to report all outcomes of redesign in healthcare.

Second, limiting the scope by only including studies that used before-and-after measurement might have led to some selection bias. Nonetheless, limiting the search strategy did ensure a solid basis for comparison of the effects of the redesign interventions.

Third, since the terminology used to describe the interventions varies greatly, we could have missed some relevant studies. We circumvented this problem by searching multiple databases with database-specific headings like MeSH terms and amplifying the strategy by searching with free-text words.

Fourth, the SQUIRE guidelines might not be the only instrument for assessing excellence in reporting. Although they were specifically developed to assess reporting excellence for this type of studies, the checklist does not provide a value judgment on the methodology (or strength of evidence) of the studies [11]. Nonetheless, by covering methodological components, the SQUIRE checklist gives a sense of the methodological strengths of a study.

Finally, using the IoM dimensions of quality of care might have made it difficult to compare findings across studies. Since the IoM does not specify which outcome measures belong to the six dimensions, there is room for interpretation. Even though this might have influenced the presentation of findings in this review, using the IoM dimensions facilitated classification of the outcomes, thereby revealing gaps in the research literature.

## Conclusion

Scientific evidence supporting process redesign in healthcare is limited and inconsistent. Outcome measures for the effect of redesign interventions vary across studies to the extent that it is impossible to draw conclusions about the impact on overall quality of care, or even on some of its dimensions. The findings of this systematic review suggest that the evaluation of process redesign interventions should be improved to reveal their full effect. It should meet the basic standards for reporting (SQUIRE guidelines) and apply more robust research designs. The influence of process redesign on patient-centered care, equity of care and timeliness warrants further research, applying outcome measures that capture the full scope of quality of care. Current research tends to ignore the long-term effects of process redesigns. Robust evaluations of their implementation should also identify the mechanisms through which effects were realized. This would help researchers and policymakers determine the value of specific interventions and offer an overview of improvement efforts that is less fragmented.

## Appendix 1

**Table 5:** Search terms used

PubMed: Medical Subject Heading (MesH) terms
(((("Organizational Innovation"[Mesh] OR "hospital restructuring"[MeSH Terms] OR "Health Care Reform"[Mesh]) AND ("Delivery of Health Care"[Mesh] OR "Health Care Sector"[Mesh])) AND ("Institutional Practice"[Mesh] OR "Clinical Protocols"[Mesh] OR "Physician's Practice Patterns"[Mesh] OR "Nurse's Practice Patterns"[Mesh])) AND ("Quality Improvement"[Mesh] OR "Quality of Health Care"[Mesh] OR "Health Care Quality, Access, and Evaluation"[Mesh] OR “Efficiency, Organizational” [Mesh] OR “total quality management” [Mesh] OR “patient safety” [Mesh] OR “patient-centered care” [Mesh]))
PubMed: Free-text words
((redesign*[Title/Abstract]OR restructur*[Title/Abstract] OR “process improvement” [Title/Abstract]) AND healthcare [Title/Abstract](AND routin* [Title/Abstract] OR process* [Title/Abstract]) AND (“quality of care” [Title/Abstract] OR “efficien*” [Title/Abstract] OR “safe*” [Title/Abstract] OR “timel*” [Title/Abstract] OR “effective*” [Title/Abstract] OR “patient-centered” [Title/Abstract] OR “equitable” [Title/Abstract])
CINAHL: CINAHL Headings terms
((MH "Work Redesign") OR (MH "Health Care Reform") OR (MH "Organizational Change") OR (MH "Organizational Restructuring”)) AND (MH "Health Care Delivery") AND ((MH "Medical Practice") OR (MH "Advanced Nursing Practice") OR (MH "Professional Practice, Research-Based") OR (MH "Professional Practice, Theory-Based") OR (MH "Nursing Practice") OR (MH "Professional Practice, Evidence-Based") OR (MH "Nursing Practice, Theory-Based") OR (MH "Nursing Practice, Research-Based") OR (MH "Nursing Practice, Evidence-Based") OR (MH "Medical Practice, Research-Based") OR (MH "Medical Practice, Evidence-Based") OR (MH "Nursing Care") OR (MH "Practice Patterns")) AND ((MH "Quality of Health Care") OR (MH "Quality Management, Organizational") OR (MH "Quality Assessment") OR (MH "Quality Improvement") OR (MH "Quality Assurance") OR (MH "Quality of Nursing Care") OR (MH "Patient Safety") OR (MH "Organizational Efficiency") OR (MH "Patient Centered Care"))
CINAHL: Free-text words
(redesign* OR restructure* OR “process improvement”) AND healthcare AND (routin* OR proces*) AND (“quality of care" OR efficiency OR safe* OR timel* OR effectiveness OR “patient-centered” OR equitable)
Web of Science: Free-text words
(redesign* OR restructure* OR “process improvement”) AND healthcare AND (routin* OR proces*) AND (“quality of care" OR efficiency OR safe* OR timel* OR effectiveness OR “patient-centered” OR equitable)
Business Premier Source: Thesaurus terms
(((((DE "REENGINEERING (Management)") OR (DE "PROCESS optimization")) OR (DE "ORGANIZATIONAL change")) AND (DE "MEDICAL care")) AND (DE "ORGANIZATIONAL effectiveness"))
Business Premier Source: free-text words
(redesign* OR restructure* OR “process improvement”) AND healthcare AND (routin* OR proces*) AND (“quality of care" OR efficiency OR safe* OR timel* OR effectiveness OR “patient-centered” OR equitable)
